# Barriers to accessing and utilising under-five primary health care services in Vhembe District

**DOI:** 10.4102/phcfm.v16i1.4429

**Published:** 2024-06-26

**Authors:** Livhuwani Tshivhase, Idah Moyo, Sophie M. Mogotlane, Sophy M. Moloko

**Affiliations:** 1 Department of Nursing, School of Health Care, Sefako Makgatho Health Sciences University, Pretoria, South Africa; 2 Department of HIV Services, Faculty of Population Solutions for Health, College of Human Sciences, Harare, Zimbabwe; 3 Department of Health Studies, University of South Africa, Pretoria, South Africa

**Keywords:** access, barriers, guardians, primary health care, under-five child

## Abstract

**Background:**

Sub-Saharan Africa continues to be the region with the highest under-five mortality rate globally, with 74 deaths per 1000 live births. Even though under-five child primary health care (PHC) services are free in South Africa, accessing such services remains challenging. Children under 5 years reportedly die from common illnesses such as pneumonia, diarrhoea and malaria, which are treatable in PHC facilities.

**Aim:**

The study explored the barriers to accessing and utilising under-five PHC services in the Vhembe District.

**Setting:**

The study was conducted in two PHC centres in Vhembe District among guardians accessing care for under-five child health services.

**Methods:**

An interpretative phenomenology design was followed using a semi-structured individual interview guide. Sixteen participants were purposively sampled for the study. Colaizzi’s steps of data analysis were followed, and trustworthiness as well as ethical principles were ensured throughout the study.

**Results:**

Four themes emerged as health system barriers, health personnel-related behaviours, health facility infrastructure barriers and guardians-related barriers. Subthemes emerged as distance from the facility, lack of resources, long waiting times; poor time management, lack of commitment and work devotion, insufficient waiting space; challenges with water and sanitation, guardians’ healthcare beliefs and the urgency of the illness.

**Conclusion:**

It is imperative that an enabling professional and friendly environment is created to facilitate better access to PHC services for children under 5 years.

**Contribution:**

The study’s findings brought insight into considering the context of the guardians in improving quality care for under 5 years.

## Introduction

Children under 5 years are reportedly dying from preventable and treatable diseases such as measles, malaria, pneumonia, diarrhoea and human immunodeficiency virus (HIV) and acquired immunodeficiency syndrome (AIDS) related diseases.^1^ These diseases are manageable at the primary health care (PHC) facilities using the Integrated Management of Childhood Illness (IMCI) strategy. The strategy integrates available measures to promote health and prevent illnesses through growth monitoring, vitamin A supplementation, immunisations, breastfeeding promotion, homecare counselling and deworming. Childhood diseases are managed through an algorithm for assessing, classifying, treating and rehabilitating.^2^

The IMCI strategy is crucial in improving child health and reducing child mortality rates. Therefore, it is essential to prioritise healthcare access for children under 5 years to enhance their quality of life. Access to healthcare provides multiple benefits, such as disease prevention, early detection, diagnosis, treatment, avoiding preventable long-term illnesses and deaths and overall physical, social and mental health and well-being of citizens.^3^ Despite the benefits, there are several barriers to accessing child health services; thus, identifying and overcoming any barriers are significant in achieving sustainable development goal (SDG) 3.

A study conducted in Malawi identified several barriers to accessing healthcare services for children under 5 years. These barriers included a lack of medicines and supplies, long waiting times, delayed facility opening times, negative attitudes of health workers, inadequate examination of sick children, long distances to health facilities and the cost of healthcare.^4,5^ Additionally, Govender^6^ reported that medicine shortages are a significant barrier to child healthcare. Supporting this statement, Rafi et al.^7^ stated that access to essential medicines is a basic human right, and the absence of medicines could infringe on a child’s right to life.

In addition, Adedini et al.^5^ found that poor attitudes of healthcare workers were significant barriers to accessing child healthcare services. Many people preferred to travel long distances to seek care at faraway healthcare facilities because they believed the quality of care provided there was superior. The study also revealed that some caregivers chose to consult faith-based healing (FBH) and traditional healers (TH) for child healthcare instead of healthcare facilities. They believed the child was suffering from an African illness that required traditional rather than Western medicines. This indicates that some guardians may not consult healthcare workers for child healthcare services but instead turn to FBH and TH.

Difficulties accessing healthcare facilities for children under 5 years can lead to missed vaccinations, leaving them vulnerable to vaccine-preventable diseases and increasing their risk of contracting infectious diseases. According to Saso et al.,^8^ this is a significant problem in South Africa, where 15% of poor rural households live more than an hour away from clinics, and 20% live the same distance from nearby hospitals. People in these areas face challenges with expensive and unreliable transportation and poor road conditions, making it difficult to access healthcare services. The long distance between these communities and healthcare facilities renders healthcare inaccessible.

Several studies have been conducted on the accessibility and utilisation of child healthcare services in Africa. These studies revealed that guardians seek healthcare facilities for their children when they present with fever, gastrointestinal and respiratory tract infections.^9,10^ However, there is limited understanding of the barriers to accessing child healthcare services in rural Venda. Hence, the study aimed to explore the barriers experienced by guardians in accessing under-five healthcare services in Vhembe District, Limpopo province.

## Research methods and design

### Study approach and design

The study was conducted from the constructivist viewpoint using a qualitative approach to understand better the study phenomena from the participants’ perspective.^11^ Interpretative phenomenological analysis (IPA) design was utilised to gain an in-depth understanding of the lived experiences of the study participants concerning barriers to accessing under-five child PHC services. Teherani et al.^12^ and Horrigan-Kelly et al.^13^ posit that this type of methodology aims to explore how people experience, uncover new meanings and understand phenomena under study. According to Creswell and Creswell^14^ and Smith and Osborn,^15^ interpretative phenomenology enables participants to narrate their stories from their lived experiences. The researcher’s choices for the approach were to ensure that they could explore, probe and gain a rich understanding of barriers to accessing healthcare through the voices of guardians of children under 5 years. This approach tallies our study’s aim and constitutes our epistemological stance.

### Study setting and participants

The study was conducted in two PHC centres of Vhembe District, Limpopo province. The district is based in the rural areas of Limpopo province. The two healthcare facilities were selected based on the high headcount of children under 5 years. These facilities provide under-five healthcare services daily, including weekends. Sixteen participants comprising grandmothers and mothers of children under 5 years accessed from the two healthcare centres in Vhembe District were recruited to participate in this study. The participants were purposefully and conveniently selected because they were the ones taking care of the children and were available at the facilities during data collection. The study included guardians of children under 5 years who were bringing a child for any child health services in the selected facilities and were willing to participate. Those who brought very ill children were excluded. Table 1 presents the demographic data of the participants.

**TABLE 1:** Biographic data for participants.

Number	Pseudonyms	Gender	Age (years)	Relationship with the child	Age of child	Number of under-five in household
1	Portia	F	29	Mother	9 months	2
2	Mashudu	F	39	Mother	1 year	5
3	Provide	F	22	Mother	4 years	2
4	Mulalo	F	28	Mother	3 years	2
5	Elisa	F	67	Grandmother	2 years	3
6	Livhuwani	F	32	Mother	3 years	3
7	Lorraine	F	56	Grandmother	6 months	1
8	Mawande	F	37	Mother	3 months	2
9	Jane	F	34	Mother	3 years	2
10	Ndivhuwo	F	28	Mother	3 years	1
11	Kgomotso	F	27	Mother	3 years	2
12	Thandi	F	28	Mother	6 weeks	2
13	Mpho	F	33	Mother	3 years	1
14	Memory	F	22	Mother	1 year 7 months	2
15	Christabel	F	27	Mother	6 months	2
16	Muofhe	F	65	Grandmother	2 years	2

### Data collection

The author (S.M.M.) conducted a pilot study and subsequent interviews. The pilot study was conducted with three guardians who were not part of the main study. This process was vital in testing the feasibility of the study and the clarity of the research questions, resulting in minor adjustments. Following the pilot, the author arranged an appointment for data collection with the facility managers. Participants were recruited while waiting for the service in the facility. The study’s purpose was explained, and those who consented to participate were interviewed in a private room provided by the manager. The interviews were audio-recorded with participants’ permission, as recommended by Van Manen.^16^ The author was unknown to the participants and was not involved in providing healthcare services to the study site. Interviews used open-ended questions in line with IPA; for example, what have been your experiences as you accessed under-five child PHC services? What were the experienced challenges in accessing under-five PHC services in Vhembe District facilities? Participants were encouraged to explore and reflect on their experiences and the meaning attached to them; this aligns with the phenomenological origins and principles of IPA articulated by Alase.^17^ All interviews were anonymised. Pseudonyms were used to address the participants. The author maintained reflexivity by engaging in self-reflection and ruling out any possible influence on participants’ expressions and interpretations. The interviews lasted 60 min or more.

The sample size of 16 participants was determined by saturation, which according to Korstjens and Moser^18^ refers to the point at which the data collection process fails to yield new information relevant to the phenomenon under study. Data saturation was attained (at the 13th participant), and three additional participants were interviewed before closure was reached at 16 participants. The sample size of 16 was adequate and justifiable for this study, as highlighted by Alase^17^ and Creswell and Creswell,^14^ who posit that in an IPA study, a sample of 2–25 is suitable for such a study. Data collection occurred between 27 June 2022 and 31 August 2022, guided by an interview guide (see Online Appendix 1).

### Data analysis

All audio-recorded interview data were transcribed verbatim into written text. Two researchers analysed the transcripts independently using an IPA framework.^15^ On the other hand, a third person acted as an independent co-coder and conducted the open coding of each transcript. Each researcher read each transcript several times and listened to the audio recording a few times. The following steps, as outlined by Smith and Osborn,^15^ guided the analysis process: (1) reading and rereading the transcript; (2) note-taking and developing emergent themes; (3) clustering the emergent themes; (4) crafting a master table of themes – composed of superordinate themes, subthemes and extracts from the interviews; (5) examining and comparing the similarities between the master tables of the themes and (6) compiling a single master list – composed of themes and subthemes. Following this, the researchers proceeded further towards interpretation and deepening the analysis. After that, the research team met to compare and discuss their master table of themes. The team had a consensus discussion and agreed on the final master table, composed of themes, subthemes and associated extracts from the transcripts (Table 2), as highlighted by Creswell and Creswell.^19^

**TABLE 2:** Themes and subthemes.

Themes		Subthemes
1.	Health system barriers	1.1 Distance from the facility 1.2 Resource constraints 1.3 Long waiting times
2.	Healthcare personnel-related behaviours	2.1 Poor time management 2.2 Lack of dedication to their work
3.	Health facility infrastructural barriers	3.1 Uncomfortable and unconducive waiting area 3.2 Challenges with water and sanitation
4.	Guardians-related barriers	4.1 Guardian healthcare beliefs 4.2 Urgency and severity of the illness

### Trustworthiness

The trustworthiness of the study was ensured through the four criteria as outlined by Lincoln and Guba^20^ cited in Nowel et al.,^21^ which are credibility, confirmability, transferability and dependability. Credibility was ensured through member checking, wherein the participants were phoned to confirm whether the findings were confirming what they voiced out during interviews. An independent coder was used to code data independently, and a consensus was reached in developing themes for the study. A thick description of the methodology, including the context for the study, was done to ensure transferability. To ensure dependability, the research process was logically documented.

### Ethical consideration

Sefako Makgatho Health Sciences University granted ethical approval for the investigation (ethical clearance number: SMUREC/H/334/2021:IR). Before collecting data, permission was obtained from the Department of Health, Limpopo province, Vhembe District, and the facility managers of the two facilities. Before data collection, verbal and written consent from participants was obtained. Confidentiality and anonymity were ensured by utilising pseudonyms when addressing the participants.

## Results

### Sociodemographic characteristics of the guardians

Participants for this study were guardians of children under 5 years who were receiving under-five child healthcare services from the two selected PHC centres. Table 1 presents the demographic data of participants.

### Barriers to accessing and utilising healthcare services by guardians of children under 5 years

Four themes and nine subthemes emerged from data analysis: health system barriers, healthcare personnel-related behaviour and health facility infrastructural and guardians-related barriers. Table 2 presents the themes and subthemes developed from the study findings.

#### Health system barriers

Participants in our study expressed challenges that hinder access to and utilisation of child health services. These challenges include distance from facilities, lack of resources and long waiting times.

**Distance from facilities:** Participants stated that although the healthcare facilities are distant from their homes, they have no option but to take their children to the facilities due to their medical needs:

‘I always feel demotivated to come here because it is far, but then there is nothing I can do about it because I need help; the 8-year-old needs medication every month, and the 6-month-old must also come for immunisation. There is also no other clinic I can go to because they are far from where I stay.’ (Jane, 34 years, mother)

‘I was told to go to B hospital, which is very far, but I went there because I did not have a choice.’ (Portia, 29 years, mother)

**Resource constraints:** Participants in the study complained about inadequate resources at the facility, specifically a shortage of nurses and medication. Some had to leave without receiving care due to a lack of available staff and medicine:

‘The government officials should be involved so that they can be told what we face here daily, and if a shortage of nurses may be the cause, then they increase the number.’ (Elisa, 67 years, grandmother)

‘It is the same thing because we sometimes find that there is no medication, which makes many people disappointed; they just leave without even consulting.’ (Mawande, 37 years, mother)

**Long waiting times:** Participants indicated that the service in the facilities is very slow, causing them to stay long in the facility. They attributed the long waiting times to a shortage of nurses, as only one nurse will be consulting children daily:

‘… [*I*]t is just that sometimes you may come and find that there are many people, and the staff is few and spend a long time waiting for the nurse to consult us one by one, that one nurse who is around.’ (Lorraine, 56 years, grandmother)

‘What can be improved is the waiting time, they are slow, the service is slow, it takes a long time to get to be assisted.’ (Livhuwani, 32 years, mother)

#### Healthcare personnel-related behaviours

Participants identified barriers from healthcare personnel behaviour, including poor time management and lack of dedication to their work.

**Poor time management:** Participants expressed concern about healthcare providers starting work late, as they must stay in the facilities for long periods and risk their children contracting diseases due to prolonged exposure:

‘Sometimes we come here early, and we only leave late, and we are women with other children to take care of, sometimes kids get illnesses from here, the service is bad.’ (Kgomotso, 27 years, mother)

‘They do not treat us well, we come here at 8 am, and you will find that they only start attending to us late.’ (Ndivhuwo, 28 years, mother)

In addition, the participants expressed concerns about waiting for healthcare workers to return from lunch as they were left unattended due to all healthcare workers taking lunch simultaneously:

‘They are very slow here, and when they go to lunch, they all leave us unattended, and they do not even consider that there might be a really sick child that could need quick medical attention.’ (Thandi, 28 years, mother)

‘I think they should change how they take their lunch; others should remain while others go for lunch.’ (Mawande, 37 years, mother)

**Lack of dedication to their work:** Participants expressed concern that healthcare workers did not value their clients and lacked dedication. They were observed using mobile phones and chatting on WhatsApp while clients waited to be serviced:

‘I mean, I brought a child having an eye problem, which looks like she had slept on her eye. I told them that the child was in pain and crying. They send me back in the line. Then I took my child and went to the hospital’s eye department. They found that the child was bitten, and they treated her there.’ (Mashudu, 39 years, mother)

‘Some of you find them on WhatsApp or Tik Tok during working hours and not attending to patients.’ (Thandi, 28 years, mother)

‘I feel drained at the thought of coming here; they should change the nurses; the nurses here really like their phones, especially WhatsApp; they attend to you while on WhatsApp or while on calls with people.’ (Muofhe, 65 years, grandmother)

#### Health facility infrastructural barriers

Participants reported that the facilities’ infrastructure was not suitable for their health. They found it uncomfortable, unconducive to their health and lacked water and sanitation.

Uncomfortable and unconducive waiting area: Participants reported that the facilities are always overcrowded and there are not enough chairs, forcing some to stand outside in bad weather, discouraging them from using the facilities. The participants had this to say:

‘The waiting area has not enough chairs for everyone for us to come and use the facilities.’ (Christabel, 27 years, mother)

‘I feel bad because we wait outside and that we might catch a cold and get pneumonia in bad weather as we wait for space in the clinic.’ (Mashudu, 39 years, mother)

**Challenges with water and sanitation:** Participants expressed concern about the poor conditions of the facilities, which included a lack of clean running water and inadequate ablution facilities that were deemed harmful to children’s health:

‘Sometimes we come here early, and we only leave late, and we are women with other children to care for; sometimes, kids get illnesses from here, and the service is terrible. The toilets are sometimes not clean.’ (Thandi, 28 years, mother)

‘There should always be medication available for patients, toilets should always be cleaned, and the taps for running water should be fixed.’ (Christabel, 27 years, mother)

#### Guardians-related barriers

Guardians’ healthcare beliefs were found to influence the utilisation of the health service. The urgency and severity of illness further influenced their utilisation.

**Guardian healthcare beliefs:** Participants have beliefs about which illnesses can be treated with traditional or Western medicine. They seek healthcare for fever and cough but not for conditions believed to be of African origin, which influences their healthcare utilisation:

‘I only come to the clinic for fever, severe cough, other diseases like *gokhonya* [*traditionally named wart-like in the female genitalia*] and Goni (traditionally named sores like over the infant’s occiput) are treated by traditional healers.’ (Kgomotso, 27 years, mother)

‘I believe that not all illnesses need Western medicine; some need traditional healers. I came when the child was to be immunised. For other African diseases, I go to the traditional healer for the family.’ (Mulalo, 28 years, mother)

**Urgency and severity of the illness:** Some guardians mentioned that they seek health based on how urgent and severe the illness is. They prefer not to take the child with mild diseases to health facilities. They only utilise the service when the child presents with severe symptoms like a high fever:

‘It influenced me in a bad way, in such a way that whenever my child has flu, I do not go to the clinic, I go and buy from the pharmacy.’ (Memory, 22 years, mother)

‘I usually do not go to the clinic when the child has a mild illness; I believe some illnesses can heal without getting to the clinic treatment. I run when a child is very hot or when the child is crying non-stop.’ (Mpho, 33 years, mother)

## Discussions

The study explored barriers to accessing and utilising child health services from the guardians’ perspective. The findings revealed multiple barriers related to the health system, healthcare providers’ behaviour, facility infrastructure and guardians’ perceptions of healthcare needs. While distance from the facility was challenging for participants, it did not affect their service utilisation, as they recognised the importance of child health treatment and immunisation services. Similarly, a study conducted in Somalia found that distance from the facility was not an essential consideration for access to the facility.^22^ In contrast, several studies found that distance from the facility was a barrier to accessing healthcare services, mainly due to a lack of reliable transportation and cost.^23,24,25^ Consequently, caregivers observed their children’s condition at home, hoping for improvement.^26^

The participants in this study felt discouraged when they had to utilise the healthcare facilities due to a lack of resources, mainly medication and nurses. They expressed the disappointment of coming to the facility and leaving without treatment. Regardless of the IMCI strategy that recognises the importance of using home remedies and essential medication, the findings show that the basic medication required to treat childhood illnesses is not always available. The medication shortage was not unique to our study; it was reported to negatively affect the quality of health services in Somalia and Malawi.^22,23^ The lack of medical supplies affects children’s health outcomes, potentially increasing the mortality rate.

In addition to a medication shortage, the lack of healthcare providers, particularly nurses, was a challenge. It is common for nurses to provide child health services in PHC settings. Similar to our findings, studies in other countries found nurses insufficient to service the number of children requiring it.^22,23^ This has led to long waiting times, negatively affecting the utilisation and satisfaction of the service. Similar findings were indicated in a study conducted in PHC facilities of Gauteng province. Clients seen complained about the long waiting times they were subjected to while in need of care.^27^ Consequently, this affects the choice of service, where facilities with short waiting periods are preferred over those with long waiting times.^22^

Our findings showed that nurse shortages did not solely cause long wait times but also poor time management by healthcare providers. Participants found the service unacceptable, as they had to wait for extended periods before receiving care, often due to healthcare workers starting work late. The lack of time management was also reported in Pakistan, where nurses were found to spend most of their time gossiping.^28^ Late commencement of work was regarded as inconsiderate of children’s conditions, exposing them to further illness. Furthermore, participants had to wait again for healthcare workers while they were all on lunch, indicating a lack of planning and flexibility from healthcare workers. Consequently, guardians felt it better to stay home than bring ill children to facilities because it could take the whole day to be attended to.

Furthermore, the healthcare providers in our study lacked dedication to their work. They spend most of their time on their phones, mainly social media. This behaviour resulted in participants feeling that they and their children were not being valued. Healthcare providers seemed uninterested in their work and inconsiderate of participants’ concerns, as reflected by sending the guardian back on the line while the child was crying inconsolably due to a painful eye. This action has resulted in the participants leaving the facility and going to the hospital for assistance. The study findings share similar sentiments with Gutierrez-Puertas et al.,^29^ which reported the use of social media to interfere with the ability to perceive the emotions of others as well as worsening communication skills with patients. Socialising at work is one of the time wasters and is detrimental to healthcare.^28^ Contrary to these findings, nurses’ use of smartphones was associated with improving their access to healthcare information, improving productivity and supporting and facilitating education for rural-based midwives.^30,31^

Poor time management and lack of dedication to healthcare service pose a significant risk to the child’s health outcome requiring urgent attention. Failure to address the challenges will become a drawback in attaining SDG 3. According to the National Department of Health,^32^ as documented in Ideal Clinic Manual, the acceptable patient waiting times in South Africa should be at most 3 h in PHC facilities. Reduced waiting time is one of the essential nonclinical attributes of health service utilisation.^21,22,23^ Therefore, all patients must receive prompt care.

Knowing that the facility is always very full, and one has to stand outside in unconducive and unfavourable weather conditions, influences the utilisation of the service. In addition, poor sanitation appeared to be a health threat for children and guardians utilising the facility. Exposure to unfavourable conditions while waiting for the service was also reported in a study conducted in South Africa. To solve the unfavourable environmental exposure, they suggested constructing rain and sun shelters outside the facilities.^33^

Traditional healers were preferred by guardians for certain childhood illnesses like ‘*gokhonya* and *ngoma*’, as they believed Western medicine from clinics or hospitals might not be effective. Guardians also tend to wait before seeking medical help, believing that some illnesses can go away on their own or be treated with over-the-counter medications from retail shops or pharmacies. Late healthcare facility visits are one result of this practice. The study findings are supported by Ngere et al.^34^ who found that caregivers prefer traditional medicines for treating childhood illnesses based on their beliefs and disease aetiology. While the diseases that could be treated with traditional medicine were not mentioned, some researchers have found that rural dwellers use traditional medicine due to its cultural acceptability, local pharmacopoeia and low cost. In contrast, urban dwellers and those with community-based health insurance are less likely to use it.^35,36^

Finally, the utilisation of child health services was further influenced by the guardian’s perception of the severity of the illness. Guardians were not using the facilities for illnesses perceived as mild and could heal without treatment. Similarly, studies elsewhere found that health services were only utilised when the symptoms were severe.^25,26^ Our study is not free of limitation as it was conducted in a healthcare facility with the possibility of involving guardians who could access the facility. A community study might give a comprehensive picture of barriers to accessing child healthcare.

## Conclusions

The study highlights the need to strengthen child healthcare systems. This can be achieved by improving health workers’ dedication and reducing waiting times. Organised healthcare services are critical to meeting clients’ needs. Healthcare facilities should be accessible, and the infrastructure should be well maintained, accommodating all users. These considerations are essential for child health programmes like the IMCI, which relies on these preferences for effective service utilisation.^22^ Communities should receive continuous health education to understand better childhood illness causes and the importance of using available services.
